# Modifications in Protein Structure and Xanthine Oxidase Inhibition of Yak Casein Induced by Protease Treatment

**DOI:** 10.1002/fsn3.4522

**Published:** 2024-10-18

**Authors:** Gongru Shang, Mingqin Deng, Yu Zhang, Huayi Suo, Jiajia Song

**Affiliations:** ^1^ College of Food Science Southwest University Chongqing People's Republic of China

**Keywords:** hydrolysates, structure, xanthine oxidase, yak casein

## Abstract

Yak milk is abundant in casein, which can generate a variety of bioactive peptides through enzymatic hydrolysis. However, the influence of enzymatic hydrolysis on the structural properties of yak casein and its inhibitory effects on xanthine oxidase (XOD) remain largely unexplored. This study demonstrated that when yak casein was subjected to treatment with flavourzyme, the degree of hydrolysis progressively increased over time, resulting in the fragmentation of the casein's flake‐like structure into smaller particles. Circular dichroism analysis revealed that after 4 h of enzymatic treatment, there was an elevation in the β‐sheet content of the yak casein hydrolysate, while other secondary structure elements diminished. Furthermore, flavourzyme treatment induced modifications in the tertiary structure of yak casein. The study also examined the impact of varying hydrolysis durations on XOD inhibitory activity, discovering that the hydrolysate obtained after 3 h displayed the highest inhibition on XOD, with an inhibition rate of (40.63 ± 3.36) %. Additionally, the fraction of the hydrolysate with a molecular weight exceeding 3 kDa demonstrated enhanced XOD inhibitory activity. This study is the first to investigate how varying hydrolysis durations with flavourzyme affect the structural characteristics of yak casein and its XOD inhibitory activity.

## Graphical abstract

The degree of hydrolysis of yak milk casein increased with hydrolysis time when treated with flavourzyme, and the secondary and tertiary structures of casein were also altered. Additionally, the yak milk casein hydrolysate exhibited the strongest xanthine oxidase inhibitory activity after 3 h of hydrolysis with flavourzyme. Modifications in Protein Structure and Xanthine Oxidase Inhibition of Yak Casein Induced by Protease Treatment.

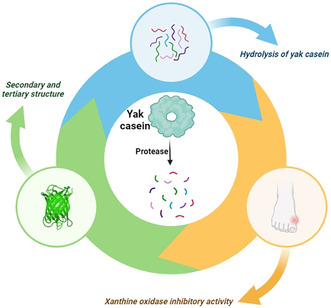

## 1 Introduction

Yak milk is renowned for its high nutritional value, encompassing proteins, essential fatty acids, vitamins, and minerals (Wang et al. 2023). Often referred to as “natural concentrated milk” (Li, Liu et al. 2023), it serves as a vital dietary staple for traditional Tibetan herders on the Qinghai‐Tibet Plateau, playing a crucial role in enhancing the health of the local populace (Guo et al. 2013). Studies indicate that yak milk has 40% to 60% more protein than cow milk, with lactoferrin levels approximately 30% higher, and nearly double the osteopontin content (Chen et al. 2021). Furthermore, the total amino acid content in yak milk surpasses that of regular cow milk, goat milk, and human milk, with essential amino acids in yak milk protein being around 45% higher in comparison to regular cow milk (Li, Liu et al. 2023). Yang et al. (2021) found that yak milk contains considerably higher levels of vitamins and minerals in comparison to regular cow milk, with variations observed at different altitudes. Luo et al. (2018) examined the lipid content and morphological characteristics of milk fat globule membranes in yak milk, finding that it contains higher amounts of cholesterol and sphingomyelin compared to regular cow milk. Additionally, subsequent studies by Luo et al. (2020) employing in vitro simulated infant gastrointestinal digestion revealed that yak milk has a considerably higher lipid digestion rate than regular cow milk, resulting in greater production of unsaturated fatty acids and displaying excellent digestive properties. Yak milk is abundant in nutrients and bioactive components, granting it numerous functional properties. These include antioxidant, anti‐tumor, anti‐fatigue, hypoxia resistance, blood pressure reduction, and cholesterol‐lowering effects. Such attributes make yak milk highly valuable for various development and application purposes (Singh, Arora, and Sarkar 2023; Wang et al. 2023). In conclusion, yak milk has high nutritional content and functional effects, and further exploration of its application value and its components is of great significance.

Casein is the predominant protein in yak milk, comprising about 60% of its total protein content (El‐Salam and El‐Shibiny 2013; Singh, Arora, and Sarkar 2023). Yak milk casein has high homology with regular cow milk casein, both having slightly higher β‐casein content and slightly lower κ‐casein content. However, yak milk casein micelles have higher hydration and colloidal calcium phosphate content, and slightly larger average size (Faccia et al. 2020). It has been reported that enzymatic hydrolysis of yak milk casein can release various bioactive peptides (Lin et al. 2018). Cheng et al. (2013) also proved that hydrolysates and their components from yak milk κ‐casein could serve as potential inhibitors against *Escherichia coli*. Liu et al. (2020) obtained natural antioxidant peptides from yak casein hydrolysate (YCH) produced by hydrolysis with alcalase and trypsin, which were more economical and safer than synthetic antioxidants. YCH also possesses radical scavenging and anti‐inflammatory capabilities and serves as a good source of anticancer peptides (Gu et al. 2022; Mao et al. 2011). Many bioactive peptides with enzyme inhibitory activity have been isolated and identified from yak milk casein, such as those inhibiting angiotensin I‐converting enzyme (ACE) (Lin et al. 2020) and dipeptidyl peptidase‐IV (Wang, Liu et al. 2021). However, the changes in the structure of yak milk casein and the inhibitory activity of xanthine oxidase (XOD) during enzymatic hydrolysis remain unclear.

Flavourzyme possesses both endopeptidase and exopeptidase activities, papain is a cysteine protease, and alcalase is a serine endopeptidase. These three proteases were selected due to their widespread use in the food industry and their distinct enzymatic specificities. In this study, the impact of hydrolysis with flavourzyme, papain, and alcalase on the degree of hydrolysis of yak milk casein was compared. Circular dichroism, fluorescence spectroscopy, and UV–visible spectroscopy were used to characterize the effects of different hydrolysis times with proteases on the secondary and tertiary structures of yak milk casein. Finally, the effects of hydrolysates and their components on XOD inhibitory activity were studied after different hydrolysis times with proteases. This study is the first to report the changes in the structure of yak milk casein and XOD inhibitory activity during hydrolysis with proteases.

## 2 Materials and Methods

### 2.1 Materials

Yak milk powder was purchased from Hongyuan Yak Dairy Co., Ltd. (Hongyuan, Sichuan, China). 8‐anilino‐1‐naphthalenesulfonic acid (ANS), Tris‐tricine‐SDS‐PAGE gel kit, protein molecular weight markers, alcalase (≥ 200,000 U/g), flavourzyme (≥ 30,000 U/g), and papain (≥ 100,000 U/g) were sourced from Solarbio Technology Co., Ltd. (Beijing, China). Xanthine oxidase and its substrate were purchased from Sigma‐Aldrich (St Louis, Missouri, USA).

### 2.2 Preparation of Yak Milk Casein

Following the method of Nguyen et al. (2020) and Zhao et al. (2020), with some modifications, 12 g of yak milk powder was mixed with 88 mL of ultrapure water and left overnight to ensure complete hydration. The reconstituted yak milk was centrifuged at 4°C, and the upper fat layer was discarded to obtain skimmed yak milk. The pH of the skimmed yak milk was regulated to 4.6 with 1 M HCl to promote casein coagulation. The mixture was then centrifuged at 4°C, and the precipitate was collected as yak milk casein. Yak milk casein was lyophilized, and its protein content was assayed using the Kjeldahl method.

### 2.3 Preparation of YCH and Ultrafiltration Fractions

Yak milk casein was hydrolyzed with alcalase, papain, and flavourzyme under optimal pH and temperature conditions (Liu et al. 2020; Zhang et al. 2023). The optimal temperature and pH for alcalase are 50°C and 11, for papain are 55°C and 6, and for flavourzyme are 50°C and 7, respectively. The ratio of yak casein to ultrapure water was 1:20 (w/v), the enzyme amount was 5% (w/w), and the hydrolysis time was 4 h. Samples were taken at 0 min (unhydrolyzed), and at 0.5, 1, 2, 3, and 4 h during the hydrolysis process. The samples underwent inactivation by being placed in a water bath at 85°C for 20 min. Once cooled, the samples were centrifuged at 4000 × *g* for 20 min at 4°C to separate the supernatant. The collected supernatant was then adjusted to a pH of 7.0 and subsequently freeze‐dried.

The sample concentration was adjusted to 10 mg/mL, and the samples were ultrafiltered using 1 and 3 kDa ultrafiltration centrifuge tubes at 4000 × *g* for 20 min. The target protein was collected from the bottom of the ultrafiltration centrifuge tubes and freeze‐dried.

### 2.4 Determination of Degree of Hydrolysis (DH) and Electrophoresis Analysis of YCH

The DH of YCH was measured based on a modified version of the ortho‐phthalaldehyde (OPA) method, as outlined by Nielsen, Petersen, and Dambmann (2006). A 400 μL YCH solution (0.1%, w/v) was mixed with 3 mL OPA reagent, allowed to stand for 2 min, and the absorbance at 340 nm was measured. The molecular weight distribution of YCH was detected based on tris‐tricine‐SDS‐PAGE electrophoresis.

### 2.5 Scanning Electron Microscopy (SEM) Analysis

Yak milk casein was hydrolyzed with flavourzyme, and YCH was obtained at 0 h, 0.5 h, 1 h, 2 h, 3 h, and 4 h, fixed on conductive adhesive, and gold‐coated. The microstructure of YCH at different hydrolysis times was observed using a scanning electron microscope at an accelerating voltage of 10 kV with a magnification of ×50 (SU‐3500, Hitachi, Tokyo, Japan) (Fang et al. 2020).

### 2.6 Structural Characteristics Analysis of YCH

#### 2.6.1 Circular Dichroism (CD) Spectroscopy Analysis

A circular dichroism spectrometer (MOS‐50010400, BioLogic, Grenoble, France) was used to scan YCH samples at different hydrolysis times (Li, Tao et al. 2023). The sample concentration was adjusted to 0.10 mg/mL, and the scanning wavelength range was set to 190–250 nm, with a scanning speed of 100 nm/min and a path length of 1 mm. Results were expressed in millidegrees (mdeg). The percentage content of each secondary structure type was analyzed using BESTSEL (https://bestsel.elte.hu/index.php).

#### 2.6.2 Fluorescence Spectroscopy Analysis

Following the method of Alizadeh‐Pasdar and Li‐Chan (2000) with modifications, the fluorescence spectra of YCH were detected using a fluorescence spectrophotometer (F‐7000, Hitachi, Tokyo, Japan). The intrinsic fluorescence spectra of YCH at different hydrolysis times were measured with a sample solution concentration of 5 mg/mL, a scanning wavelength range of 300–500 nm, an excitation wavelength of 290 nm, and both the excitation and emission slit widths set to 5 nm.

The extrinsic fluorescence spectra of YCH at different hydrolysis times were measured with a sample solution concentration of 1 μg/mL, where 1.5 mL of the sample solution was mixed with 50 μL of ANS (8 mmol/L). The scanning wavelength range was 400–700 nm, the excitation wavelength was 390 nm, and both the excitation and emission slit widths were set to 10 nm.

#### 2.6.3 UV–Visible Spectroscopy Analysis

The UV spectra of YCH were detected using a UV–visible spectrophotometer (UV‐2600/2700, Shimadzu, Kyoto, Japan) with a sample concentration of 0.10 mg/mL, ultrapure water as blank, and a scanning wavelength range of 190–800 nm.

### 2.7 Determination of XOD Inhibitory Activity

The inhibition effect of YCH on XOD was assayed using a spectrophotometric method (Thaha et al. 2021). The initial enzyme activity of the XOD stock solution was 5.92 U/mL, and an XOD working solution was prepared by diluting 100 μL of the stock solution to 30 mL. The sample solution concentration was adjusted to 1% (w/v), and the xanthine solution concentration to 0.48 mM. Fifty microliters of the test sample and 50 μL of the XOD working solution were combined and mixed for 30 s. The mixture was then left to stand at room temperature for 5 min. Subsequently, 150 μL of the xanthine solution was added, mixed for another 30 s, and left to stand at room temperature for an additional 5 min. Finally, the absorbance was measured at 290 nm using a microplate reader (Feyond‐A300, AllSheng Instruments, Hangzhou, Zhejiang Province, China).

### 2.8 Statistical Analysis

All graphs were plotted and modified using GraphPad 8 (GraphPad Software Inc., San Diego, CA, USA). Statistical and variance analyses were carried out using SPSS Statistics 23 (IBM Inc., Chicago, IL, USA). One‐way ANOVA and Tukey post hoc tests were used for statistical analysis, and a significance level of *p* < 0.05 was considered statistically significant.

## 3 Results and Discussion

### 3.1 Hydrolysis of Yak Milk Casein by Proteases

**FIGURE 1:**
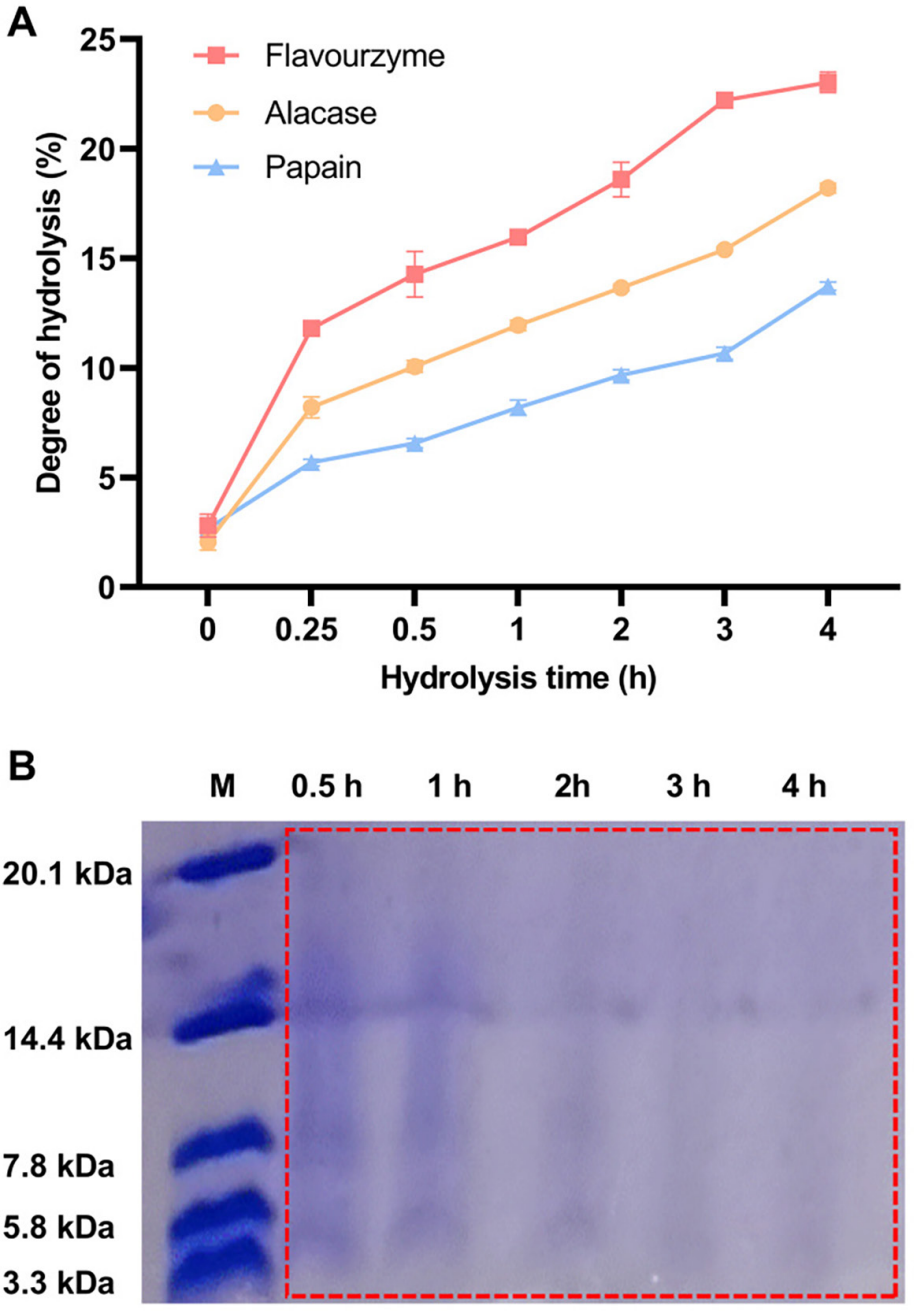
Degree of hydrolysis of yak milk casein hydrolysate. (A) Degree of hydrolysis of yak milk casein treated with different enzymes; (B) tris‐tricine SDS‐PAGE analysis of yak milk casein hydrolysate at different hydrolysis times.

This study measured the impact of different hydrolysis times with various proteases on the DH of yak milk casein. As shown in Figure 1A, yak milk casein can be hydrolyzed by flavourzyme, papain, and alcalase, with DH increasing over time. The DH of yak milk casein treated with flavourzyme was greater than that achieved with the other two proteases, reaching 23.03 ± 0.46% after 4 h of hydrolysis. Tris‐tricine‐SDS‐PAGE electrophoresis analysis of YCH at different hydrolysis times with flavourzyme showed that at 0.5 h of hydrolysis, the main bands of YCH were distributed between 3.3 and 20.1 kDa, and the bands of hydrolysis products gradually weakened with increased hydrolysis time (Figure 1B).

Yak milk, abundant in casein, serves as an excellent source for producing various bioactive peptides. Enzymatic hydrolysis of yak milk casein can yield peptides with diverse bioactivities, including antimicrobial peptides (Cheng et al. 2013; Zhang et al. 2023), antioxidant peptides (Liu et al. 2020), and angiotensin I‐converting enzyme inhibitory peptides (FitzGerald and Meisel 2007). In this study, based on the results of DH and Tris‐tricine‐SDS‐PAGE electrophoresis, flavourzyme effectively hydrolyzed yak milk casein. This is related to the fact that flavourzyme is a cysteine protease containing leucine aminopeptidase activity, possessing both exopeptidase and endopeptidase activities (Karami et al. 2022).

### 3.2 Effect of Different Hydrolysis Times on the Microstructure of Yak Milk Casein

**FIGURE 2:**
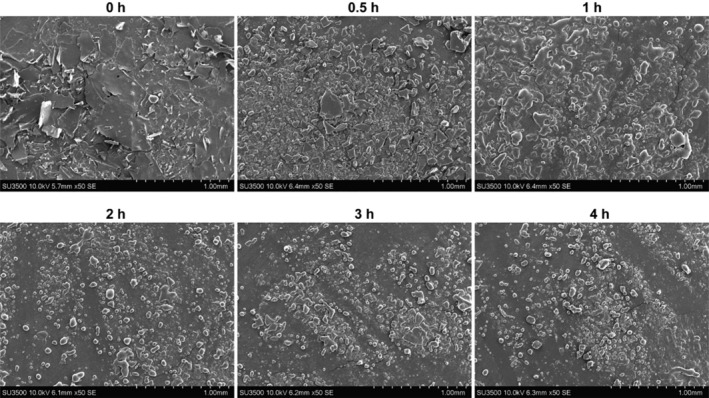
Scanning electron microscopy images of yak milk casein hydrolysate at various hydrolysis times.

The changes in the microstructure of yak milk casein hydrolyzed by flavourzyme at different times were observed using a scanning electron microscope. As shown in Figure 2, the untreated yak milk casein mostly had a complete and clear flake structure (Figure 2A), while the flake structure gradually broke into smaller particles with increased hydrolysis time (Figure 2F).

This study is the first to observe the impact of different hydrolysis times with flavourzyme on the microstructure of yak milk casein using a scanning electron microscope. The changes in the microstructure further reveal that yak milk casein can be hydrolyzed by flavourzyme to produce smaller molecular fragments.

### 3.3 Effect of Different Hydrolysis Times on the Secondary Structure of Yak Milk Casein

**FIGURE 3:**
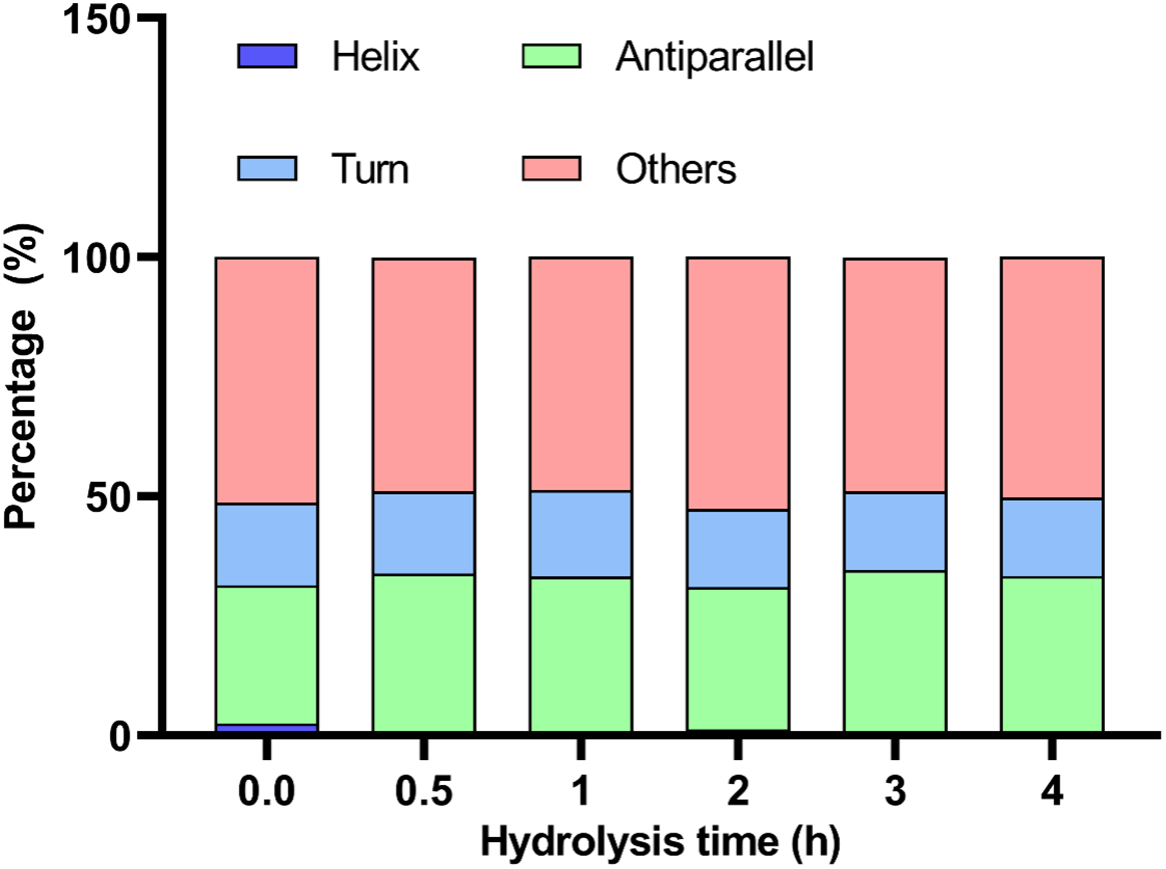
Percentage content of each secondary structure in yak milk casein hydrolysate at various hydrolysis times.

The alterations in the secondary structure of yak milk casein treated with flavourzyme were analyzed using CD spectroscopy. As illustrated in Figure 3, the percentage content of each type of secondary structure before and after hydrolysis was analyzed using the BESTSEL website. The results in the current study suggested that the untreated yak milk casein consisted of 2.5% helix, 28.9% antiparallel, 17.3% turn, and 51.3% other structures. After 4 h of hydrolysis, the antiparallel content in YCH increased to 33.3%, while the helix content decreased to 0%, and the proportions of turn and others also decreased to 16.5% and 50.3%, respectively.

In protein secondary structures, β‐sheets are considered relatively stable and compact, whereas α‐helices, β‐turns, and random coils are relatively flexible and open (Pan et al. 2022). The analysis of secondary structure percentages indicated that the α‐helix content decreased while the β‐sheet proportion increased in yak milk casein hydrolyzed by flavourzyme. These results are consistent with the structural alterations observed in lupin protein hydrolyzed by flavourzyme (Fadimu et al. 2021).

### 3.4 Effect of Different Hydrolysis Times on the Tertiary Structure of Yak Milk Casein

**FIGURE 4:**
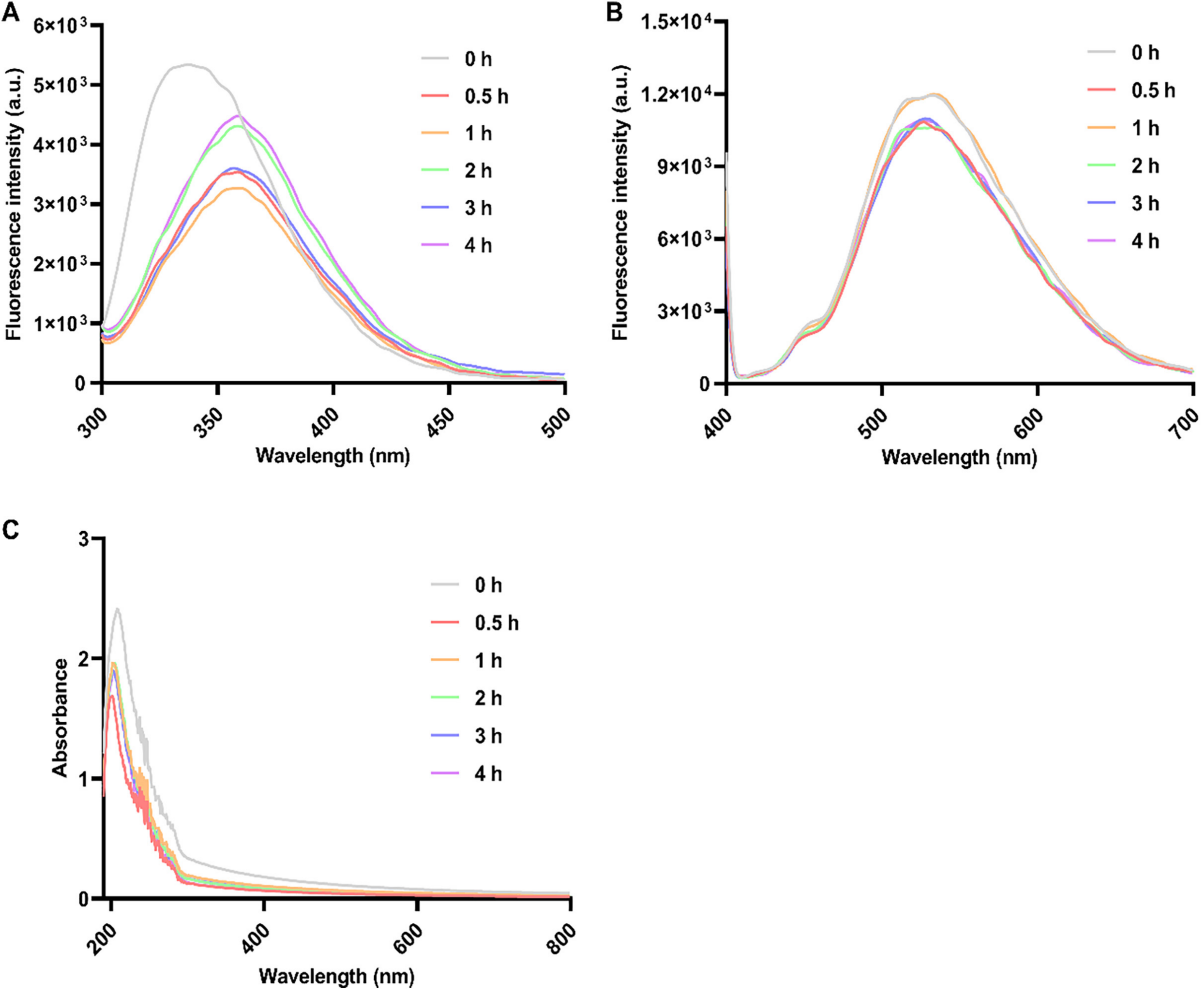
Fluorescence and UV absorption spectra analysis of yak milk casein hydrolysate. (A) Intrinsic fluorescence spectra; (B) extrinsic fluorescence spectra; (C) UV absorption spectra.

The intrinsic fluorescence spectra of yak milk casein and YCH at various hydrolysis times are presented in Figure 4A. Compared with yak milk casein, the maximum fluorescence intensity of the YCH decreased, and the maximum emission wavelength of the YCH showed a red shift from 337 nm to 358 nm. As illustrated in Figure 4B, the extrinsic fluorescence spectra indicated that the maximum fluorescence intensity of YCH treated with flavourzyme was lower than that of yak milk casein. The UV–visible spectra of YCH obtained at different hydrolysis times are displayed in Figure 4C. Our results clearly showed that untreated yak milk casein had a characteristic absorption peak at 250–280 nm. After hydrolysis with flavourzyme, the maximum absorption intensity of YCH at 250–280 nm significantly decreased compared to yak milk casein.

Intrinsic fluorescence is mainly generated by aromatic amino acids in proteins. Analyzing the changes in intrinsic fluorescence spectra of YCH can determine the impact of hydrolysis on the tertiary structure of proteins (Ai et al. 2019; Sponton et al. 2014). Typically, most aromatic amino acid residues capable of fluorescence are buried inside the protein. As the protein's spatial structure unfolds, amino acid side chains become increasingly exposed on the molecular surface, leading to a heightened polarity in the environment around the aromatic amino acids (Wang, Wang et al. 2021). The observed red shift in the maximum absorption wavelength of YCH suggests structural alterations in YCH relative to untreated yak milk casein, potentially exposing more aromatic amino acids. The maximum fluorescence intensity can be influenced by energy transfer between tyrosine and tryptophan, as well as fluorescence quenching caused by neighboring groups (Zhang et al. 2019). Thus, the reduced maximum fluorescence intensity of YCH compared to yak milk casein could be involved with conformational changes induced by flavourzyme treatment, which might decrease energy transfer from Tyr to Trp or increase the presence of fluorescence‐quenching groups.

ANS is an external fluorescence probe that attaches to exposed hydrophobic areas in certain unfolded proteins, generating fluorescence that increases in correlation with the level of hydrophobicity (Cheng et al. 2013). The lower maximum fluorescence intensity of YCH compared to yak milk casein indicated lower surface hydrophobicity of YCH, which may be involved with hydrophobic amino acids being re‐buried in the new structure after enzyme treatment or the aggregation of hydrophobic amino acids due to hydrophobic interactions (Wang et al. 2022).

UV–visible spectroscopy can qualitatively analyze structural changes in substances by observing changes in absorption peaks (Huo et al. 2019). The characteristic peaks at 250–280 nm might correspond to the aromatic amino acids (tryptophan, tyrosine, phenylalanine) in yak milk casein, including phenylalanine (257 nm), tyrosine (278 nm), and tryptophan (279 nm) (Fadimu et al. 2021). The reduced maximum absorption intensity of YCH at 250–280 nm after flavourzyme treatment may be due to some aromatic amino acid residues being reincorporated in the protein structure, consistent with the results of Ai et al.'s (2019) study on the enzymatic hydrolysis of egg white.

### 3.5 Inhibition of XOD by YCH

**FIGURE 5:**
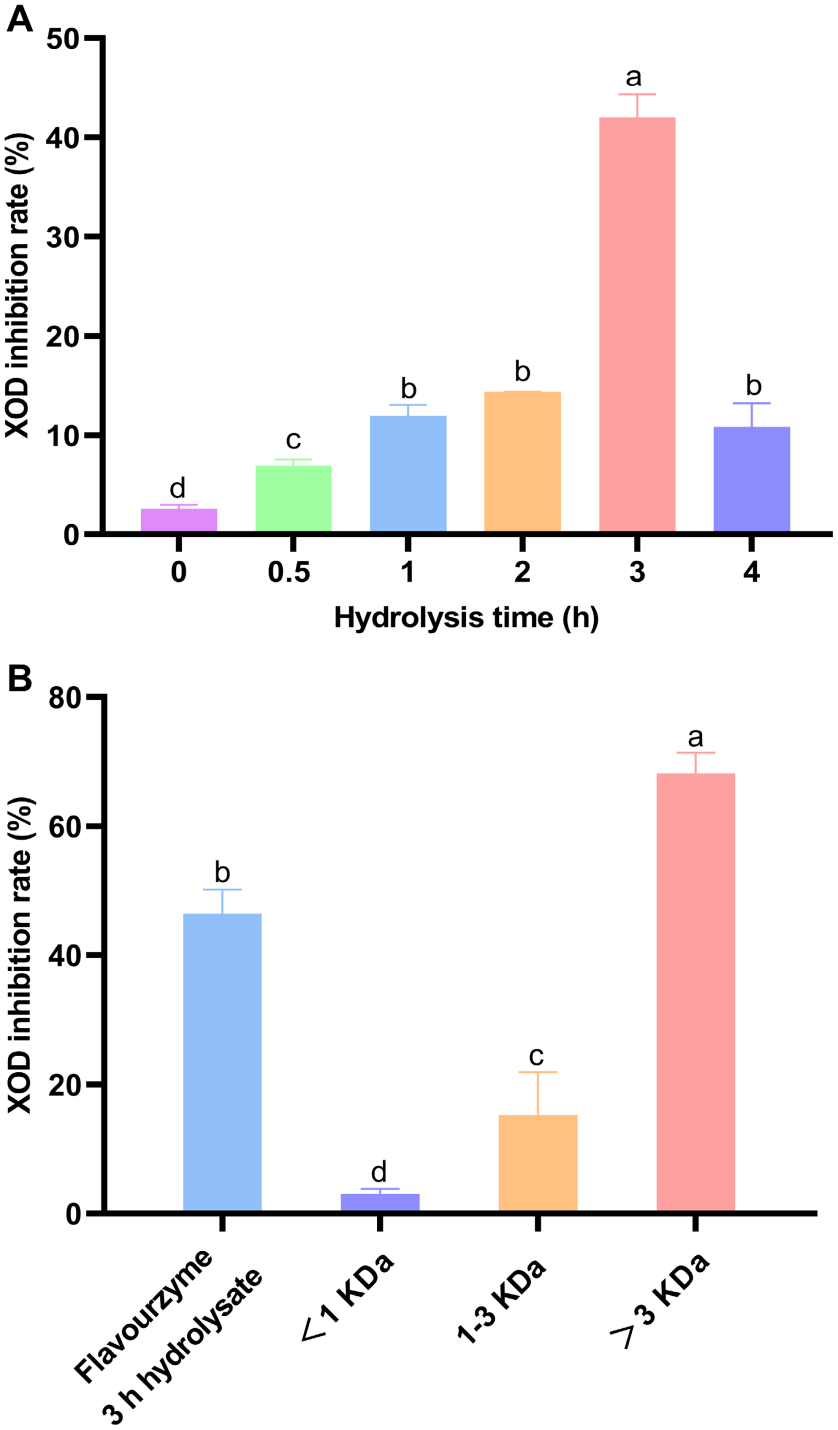
Inhibitory effect of yak milk casein hydrolysate on xanthine oxidase (XOD). (A) Inhibitory effect of yak milk casein hydrolysate on XOD; (B) inhibitory effect of different fractions of 3‐h flavourzyme hydrolysate on XOD. Different letters indicate significant differences (*p* < 0.05).

The XOD inhibitory effect of YCH hydrolyzed by flavourzyme increased with hydrolysis time within 3 h, reaching a maximum inhibitory activity of (40.63 ± 3.36) % after 3 h of hydrolysis (Figure 5A). However, the XOD inhibitory activity of YCH significantly decreased after 3 h of hydrolysis (*p* < 0.05). The 3‐h hydrolysate was further separated into fractions with different molecular weights. Figure 5B shows that the XOD inhibitory activity of fractions < 1 kDa and 1–3 kDa was lower than that of untreated yak milk casein (*p* < 0.05), whereas the XOD inhibition effect of fractions > 3 kDa was higher than that of untreated yak milk casein, with an inhibition rate of (67.00 ± 3.51) %.

Currently, food‐derived proteins from fish (Hou et al. 2022), nuts (Zhao et al. 2022), rice (Zhu et al. 2021), and whey (Qi et al. 2022) have been reported for preparing XOD inhibitory peptides. This study is the first to document the inhibition effects of YCH on XOD. The various bioactivities of hydrolyzed proteins are closely related to the degree of hydrolysis. This study's results indicate that within 3 h of hydrolysis, the XOD inhibitory activity of YCH gradually increased, but the bioactivity decreased after more than 3 h. This is similar to the study on ACE inhibitory activity of YCH, where the highest ACE inhibition was observed at 4 h of hydrolysis, decreasing after more than 4 h (Mao et al. 2007), possibly due to excessive hydrolysis further altering the structure of peptides (Mota et al. 2006). Additionally, studying the XOD inhibitory activity of different YCH fractions revealed that the inhibition decreased when the fraction was < 3 kDa, supporting the conclusion that excessive hydrolysis damages the structure of peptides in YCH, reducing XOD inhibitory activity. However, the mechanism and related amino acid sequences of yak milk casein peptides inhibiting XOD remain unclear and require further investigation.

## 4 Conclusion

The results of this study indicate that the DH of yak milk casein increases with hydrolysis time when treated with flavourzyme, and the secondary and tertiary structures of casein are also altered. Additionally, the yak milk casein hydrolysate exhibited the strongest XOD inhibitory activity after 3 h of hydrolysis with flavourzyme, with fractions greater than 3 kDa showing stronger XOD inhibitory activity. This study provides a theoretical basis for developing XOD inhibitory peptides from yak milk casein.

## Author Contributions

**Gongru Shang:** data curation (equal), investigation (equal), methodology (equal). **Mingqin Deng:** validation (equal), visualization (equal), writing – original draft (equal). **Yu Zhang:** formal analysis (equal), software (equal). **Huayi Suo:** resources (equal), writing – review and editing (equal). **Jiajia Song:** conceptualization (equal), funding acquisition (equal), project administration (equal), supervision (equal), writing – original draft (equal), writing – review and editing (equal).

## Conflicts of Interest

The authors declare no conflicts of interest.

## Data Availability

The data that support the findings of this study are available from the corresponding author upon reasonable request.
